# 0.26-Hz-linewidth ultrastable lasers at 1557 nm

**DOI:** 10.1038/srep24969

**Published:** 2016-04-27

**Authors:** Lifei Wu, Yanyi Jiang, Chaoqun Ma, Wen Qi, Hongfu Yu, Zhiyi Bi, Longsheng Ma

**Affiliations:** 1 State Key Laboratory of Precision Spectroscopy, East China Normal University, Shanghai, 200062, China

## Abstract

Narrow-linewidth ultrastable lasers at 1.5 μm are essential in many applications such as coherent transfer of light through fiber and precision spectroscopy. Those applications all rely on the ultimate performance of the lasers. Here we demonstrate two ultrastable lasers at 1557 nm with a most probable linewidth of 0.26 Hz by independently frequency-stabilizing to the resonance of 10-cm-long ultrastable Fabry-Pérot cavities at room temperature. The fractional frequency instability of each laser system is nearly 8 × 10^−16^ at 1–30 s averaging time, approaching the thermal noise limit of the reference cavities. A remarkable frequency instability of 1 × 10^−15^ is achieved on the long time scale of 100–4000 s.

Spectrally-narrow laser sources are central to many applications, such as gravitational wave detection, optical atomic clocks, precision spectroscopy and quantum computation^1^,^2^,^3^,^4^,^5^. To meet those requirements, lasers with a linewidth of a few Hz or sub-hertz have been constructed^3^,^6^,^7^,^8^,^9^,^10^,^11^. Among them, many have focused on the particular wavelength of 1.5 μm. Since it is low-loss window of optical fiber, many groups have developed narrow-linewidth ultrastable laser systems at 1.5 μm for coherent transfer of light through fiber^12^,^13^,^14^,^15^. When compact and low-cost Er:fiber-based laser frequency comb is used to transfer the coherence from 1.5 μm lasers to other optical wavelengths or microwave region^16^,^17^, it enables many novel applications such as direct frequency comb spectroscopy and low noise microwave generation^4^,^18^. Furthermore, 1.5 μm narrow-linewidth lasers could also be used to study two-electron interactions via precision spectroscopy of helium^19^.

For those attractive applications, narrow-linewidth ultrastable laser systems at 1.5 μm have been constructed based on different techniques. By frequency-stabilizing to the resonance of room-temperature high-finesse ultrastable Fabry-Pérot (F-P) optical cavities with the Pound-Drever-Hall (PDH) technique^20^, lasers at 1.5 μm with a linewidth of ∼1 Hz or sub-hertz have been constructed^14^,^21^. A 40-mHz-linewidth laser has been demonstrated by stabilizing to a silicon single-crystal cavity operated at 124 K^11^ for low thermal noise^22^. By directly phase-locking to a cavity-stabilized laser in the visible region via an optical frequency comb as a transfer oscillator, a 1 Hz-linewidth laser at 1.5 μm has been constructed^23^.

In this paper, the frequencies of two 1557 nm diode lasers are independently stabilized to two 10-cm-long ultrastable F-P cavities with the PDH technique. To achieve a thermal-noise-limited performance, each cavity, operated at room temperature, is deliberately designed for better isolation from environmental vibration and temperature fluctuation. By comparing two similar laser systems, each laser system has a most probable linewidth of 0.26 Hz and fractional frequency instability of 8 × 10^−16^ at an averaging time of 1–30 s. Compared to those using cryogenic cavities, the laser systems described in this paper are relatively simple while they still provide excellent frequency stability on both short-term and long-term scale of a few thousand seconds.

## Results

### Lasers frequency-stabilized to F-P reference cavities

**Figure 1:**
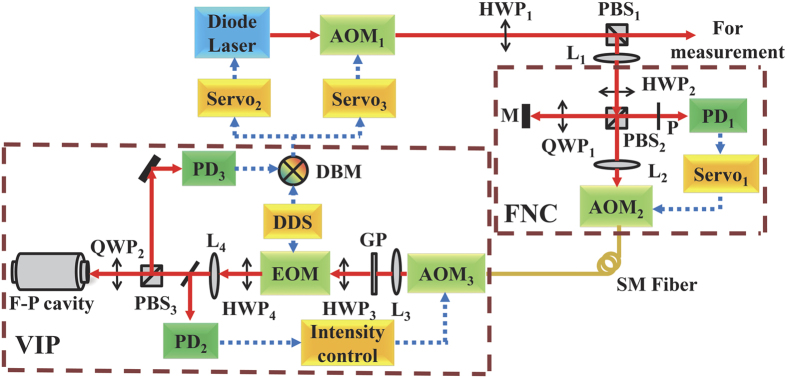
Diagram of the experimental setup. AOM, acousto-optic modulator; HWP, half-wave plate; QWP, quarter-wave plate; L, lens; M, mirror; PBS, polarization beam splitter; P, polarizer; PD, photo-detector; SM fiber, single-mode fiber; EOM, electro-optic modulator; GP, Glan polarizer; DDS, direct digital synthesizer; DBM, double balance mixer; VIP, vibration isolator platform.

The diagram of the experimental setup of one laser system is shown in Fig. 1. The laser source is a planar external cavity laser operating at 1557 nm with an output power of 10 mW. The output light is firstly frequency-shifted by +110 MHz on an acousto-optic modulator (AOM_1_), which is used as an isolator and the executor of fast laser frequency control as well. Then the first-order diffracted laser light passes through a combination of a half-waveplate (HWP_1_) and a polarization beam splitter (PBS_1_), which split the light into two beams with proper light power ratio. One light beam is sent to an F-P reference cavity while the other light beam is used for measurement. In order to isolate the F-P reference cavity from environmental vibration (see Method section), the cavity as well as the optics for the PDH technique are placed on a passive vibration isolation platform and enclosed in an acoustic isolation chamber. A 2-meter-long single-mode (SM) optical fiber is used to deliver the laser light from the laser source to the F-P cavity. Since undesirable random phase noise induced by perturbations via the SM fiber will degrade the light coherence, a fiber noise cancellation (FNC) technique is employed^24^ (see Method section).

The output of the SM optical fiber is firstly frequency-shifted by 110 MHz (+1 order) on AOM_3_, whose diffraction efficiency is adjusted by controlling the RF driving power for light intensity stabilization. Then the diffraction light from AOM_3_ is phase-modulated on an electro-optic modulator (EOM) at the modulation frequency of 50 MHz, where the laser intensity noise approaches shot noise. HWP_3_ and a high extinction-ratio Glan polarizer (GP) before the EOM are used to minimize residual amplitude modulation (RAM) of the EOM by adjusting the light polarization to match the main axial of the EO-crystal. Moreover, we controlled the temperature of the EOM slightly above room temperature with a fluctuation of 3–5 mK to reduce RAM which arises from temperature dependent EO-crystal birefringence. After the EOM, a portion of the light is detected on a DC detector (PD_2_) to monitor the light power for intensity stabilization. A combination of HWP_4_ and PBS_3_ adjust the light power incident to the reference cavity. Lenses L_3_ and L_4_ are implemented to adjust the size of light beam to match the mode of the F-P cavity. The cavity reflection is picked on PBS_3_ and is steered onto a detector (PD_3_) by PBS_3_. QWP_2_ is used to make the light maximally reflected by PBS_3_ and prevent feedback of light to the EOM and other optics in the original path. The beat note between the light carrier and modulation sidebands in the cavity reflected light is detected on PD_3_. The signal from PD_3_ is mixed with part of the driving signal of the EOM on a double balanced mixer (DBM) to obtain a laser frequency discrimination signal. This signal is then sent to Servo_2_ to control the current injected into the laser module and Servo_3_ to control the driving frequency of AOM_1_. In the experimental setup, the laser frequency servo bandwidth is 175 kHz.

### Laser linewidth

We have constructed two similar but independent laser systems at 1557 nm and tested the performance of the laser systems by beating against each other on a photodiode. The light from one laser is sent to another system for beating by optical fibers, whose random phase noise is cancelled by active cancellation (see Method section).

**Figure 2:**
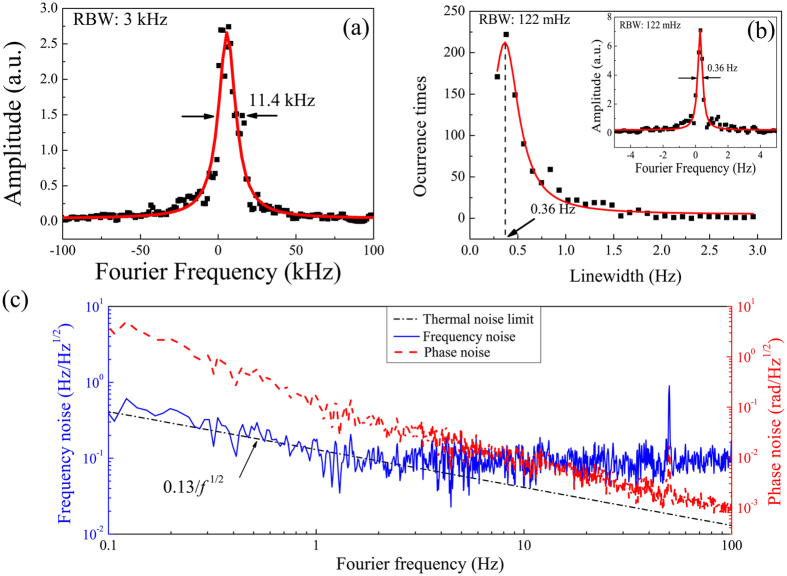
Linewidth measurements, frequency and phase noise spectral density of one laser system. (**a**) The spectrum of beat note between two free-running lasers (black squares) with 3 kHz RBW and its Lorentzian fitting curve (red solid line). (**b**) Linewidth distribution of 1000 groups of spectra (black squares) with 122 mHz RBW and Lorentzian fitting curve (red solid line). The inset shows a spectrum of the beat note between two cavity-stabilized lasers (black squares) with 122 mHz RBW and its Lorentzian fitting curve (red solid line). (**c**) The black dash dots show the calculated thermal-noise-limited frequency noise spectral density of one laser system at 0.13 Hz/*√f*. The blue solid line shows the frequency noise spectral density of one laser system and it is close to the thermal-noise-limited frequency noise at Fourier frequencies lower than 3 Hz. The red short dash line shows the calculated laser phase noise based on the relation of *S*_*v*_(*f* ) = *f*^2^*S*_*φ*_(*f* ).

To measure the laser linewidth, the beat note between two laser systems is down-converted to 50 kHz and sent to a spectrum analyzer (Agilent E4402B). As shown in Fig. 2(a), the linewidth of the beat note between two free-running lasers is as large as 11.4 kHz with a resolution bandwidth (RBW) of 3 kHz and sweeping measurement time of 70 ms. As long as the laser frequencies are tightly stabilized to the resonance of the ultrastable F-P cavities, the linewidth of the beat note is significantly reduced. For a higher resolution measurement of linewidth, a fast Fourier transform (FFT) spectrum analyzer (SR770) with input frequency range of 0–100 kHz is used. As the beat note between the two lasers is 105 MHz, it is down-converted to nearly 1 kHz and continuously measured by a computer for 10 hours. 1000 groups of spectra of the beat note were recorded. Each spectrum is measured with a RBW of 122 mHz and a measurement time of 8 s, which is fitted with a Lorentzian function to obtain the linewidth. A linear frequency drift of 0.03 Hz/s is removed during measurement by feeding forward to one of synthesizers. Figure 2(b) shows the distribution of the measurement. The most probable linewidth of the beat note between two cavity-stabilized lasers is around 0.36 Hz. The possibility of the linewidth of 0.36 ± 0.1 Hz is 54%. By assuming each laser system contributes equally to the measurement, each laser system has a most probable linewidth of 0.26 Hz.

### Laser frequency and phase noise

Besides, the laser frequency noise is measured by stabilizing one of the lasers to Cav_1_ and keeping the left portion of the laser light on resonance with Cav_2_. Using the PDH signal of Cav_2_, the laser frequency noise spectrum is obtained. Figure 2(c) shows the frequency noise spectral density of one laser system (blue solid line). It is close to the calculated thermal noise limit at 0.13 Hz/√*f* (black dash dots) at Fourier frequencies lower than 3 Hz, where *f* is the Fourier frequency. Besides, the laser phase noise spectral density S_*φ*_(*f* ) is shown with red short dash line, which is calculated with the relation of S_*v*_(*f* ) = *f*^2^S_*φ*_(*f* ).

### Frequency instability

**Figure 3:**
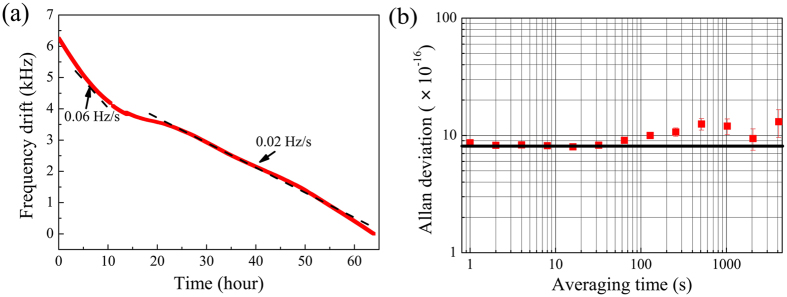
Laser frequency drift and Allan deviation. (**a**) Relative frequency drift of the beat note between two cavity-stabilized laser systems over 64 h (red solid line). The black dash lines show the frequency drift rates. (**b**) Fractional frequency instability of one cavity-stabilized laser system after a linear drift of 0.03 Hz/s is removed. The solid line indicates that the thermal-noise-limited frequency instability of 8 × 10^−16^.

The relative frequency drift between the two cavity-stabilized systems is measured by counting the beating frequency of two lasers over 64 hours (The counter is Agilent 53132A). As shown in Fig. 3(a), the total frequency drift is 6.25 kHz. The frequency drift rate is within 0.08 Hz/s. In 90% of time, the laser drifts less than 0.04 Hz/s. The fractional frequency instability of one cavity-stabilized laser is shown in Fig. 3(b) after removing a linear drift of 0.03 Hz/s during the calculation. At an averaging time of 1–30 s, the fractional frequency instability of one laser (Allan deviation) reaches the thermal-noise-limited performance of the reference cavity of 8 × 10^−16^. Benefiting from the thermal shields inserted between the FP cavity and the vacuum chamber, the laser frequency instability at an averaging time of 100–4000 s can be as low as 1 × 10^−15^.

## Discussion

In conclusion, two lasers operating at 1557 nm are independently stabilized to two F-P cavities. With good isolation of the F-P reference cavities from environmental perturbations, the reference cavities approach a thermal-noise-limited performance. The most probable laser linewidth is 0.26 Hz and the laser frequency instability is around 8 × 10^−16^ at the 1–30 s averaging time. For a time scale of 100–4000 s, laser frequency instability of 1 × 10^−15^ is achieved.

The laser systems reported in this paper is limited by the thermal noise of the reference cavities. To further improve the performance of the lasers, it is inevitable to reduce the thermal noise of the reference cavities. Either using cryogenic cavities with the coefficient of thermal expansion close to zero at low temperatures or using room-temperature cavities with longer length^7^,^11^,^25^, the thermal-noise-limited laser frequency instability will be reduced to 1 × 10^−16^. However, to keep the laser system as simple as possible, it might be a solution by using low mechanical loss mirrors and coatings^8^,^25^,^26^. For a 10-cm-long cavity with fused silica (FS) mirrors coated with crystalline coating of AlGaAs, the thermal-noise-limited laser frequency instability will approach 3 × 10^−16^.

We are transferring the coherence of the 0.26 Hz-linewidth laser light to remote locations through fiber links for potential applications such as precision spectroscopy and low noise microwave generation at remote ends.

## Methods

### Fiber noise cancellation

In the FNC, the fiber phase noise is detected by comparing the fiber round-trip light against the local reference light on a Michelson interferometer. As shown in Fig. 1 combination of HWP_2_ and PBS_2_ splits light into two interferometric arms with suitable intensity ratio. The light in the short reference arm goes through a quarter-wave plate (QWP_1_) and is reflected by a high reflectance mirror. Then it passes through QWP_1_ again and goes through PBS_2_. The light in the long arm is frequency-shifted by +77.5 MHz on AOM_2_ and is coupled into the SM optical fiber. At the remote end of the SM fiber, a small portion of the light is reflected by the flat surface of the SM fiber at the remote end and passes through AOM_2_ again. Both light beams from the two arms are combined on PBS_2_ and beat against each other on a photo detector (PD_1_). The beating signal of 155 MHz is mixed with a 155 MHz local oscillator to extract the fiber phase noise. This signal is used as an error signal to compensate the fiber phase noise by tuning the driving frequency of AOM_2_. In order to protect the optics from air flow, both the laser and optics for the FNC system are sealed in an acrylic box.

### F-P cavities

The dominant limitation of the laser frequency instability is usually the length instability of F-P cavity.

To reduce the thermal expansion of the F-P cavities, each F-P cavity is made of ultralow expansion (ULE) glass, whose coefficient of thermal expansion (CTE) is within ±30 ppb/K. Two mirrors made of ULE glass with curvature of *R*_1_ = ∞ and *R*_2_ = 0.5 m are optically-contacted to a 10-cm-long cylinder spacer. To reduce the thermal sensitivity, each cavity is located in two layers of thermal shields as low-pass thermal filters. The inner layer is made of gold-plated copper while the outer layer is made of high-polished aluminum for less weight. The reference cavity as well as two layers of thermal shields is enclosed in an aluminum vacuum chamber evacuated to 5 × 10^−7^ torr. This kind of configuration is calculated to have a thermal time constant of 28.8 hours^27^, which helps to reduce the thermal sensitivity of the reference cavity to environmental thermal fluctuation with a period of 1 hour by three orders of magnitude when compared with the system without any thermal shield. Then the vacuum chamber is enclosed in thermal isolations and temperature-stabilized above room temperature with a fluctuation of <2 mK. The vacuum chamber and the optics for the PDH technique are on the vibration isolator platform, which resides in a box made of aluminum alloy plates covered with acoustic absorption foam. The box protects the cavity from air flow, temperature fluctuation and acoustic noise. Two F-P reference cavities are independently placed on two passive vibration isolator platforms and enclosed in two acoustic isolation chambers.

**Figure 4:**
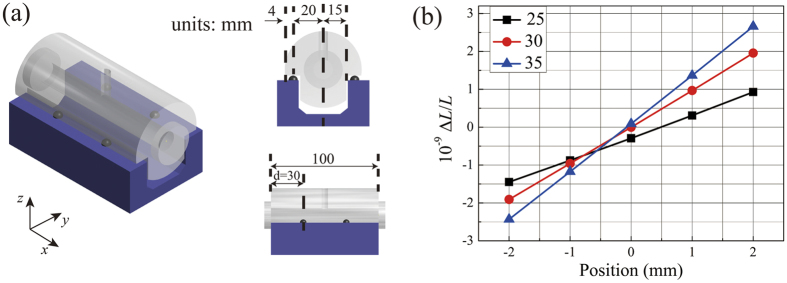
Shape of cavity and calculation results. (**a**) Three-dimensional view of the reference cavity and its support. The cavity is placed on a U-shape block and supported by four hemisphere-rubbers which are 30 mm away from the ends of the cylinder spacer along the optical axis. (**b**) The vibration sensitivity of probe points on mirrors of the reference cavity along *z* axis to accelerations along *z* axis when *d* varies according to finite element analysis. Probe points at the position of 0 mm are at the center of the mirrors.

In order to reduce the vibration sensitivity of the F-P cavities, the cavity supporting configuration is designed according to finite element analysis with ANSYS. Figure 4(a) shows the three-dimensional view of the cavity. The F-P cavity is horizontally mounted on four hemisphere rubbers, which are sitting on a U-shape fused silica block. Figure 4(b) shows the simulated sensitivity of the cavity length to an acceleration of g = 9.8 m/s^2^ along *z* axis. When the hemisphere rubbers are 30 mm away from the ends of the cylinder spacer along the optical axis (*x* axis), the cavity has the smallest vibration sensitivity of 5.5 × 10^−13^/g at the center of the mirror.

The finesse of the cavities is measured to be ∼270,000 for Cav_1_ and ∼250,000 for Cav_2_ from frequency-sweep optical-heterodyne cavity-ring-down spectroscopy^28^, corresponding to a cavity linewidth of ∼5.6 kHz and ∼6 kHz, respectively. The cavity reflection contrasts are 67% for Cav_1_ and 60% for Cav_2_, respectively.

## Additional Information

**How to cite this article**: Wu, L. *et al.* 0.26-Hz-linewidth ultrastable lasers at 1557 nm. *Sci. Rep.* **6**, 24969; doi: 10.1038/srep24969 (2016).
